# Applying the unattainable triangle in cardio-oncology care: Balancing cost, quality, and time

**DOI:** 10.18632/oncotarget.28738

**Published:** 2025-06-04

**Authors:** John Hoverson, Chirag Buch, Juan Ulloa-Rodriguez, Samuel Governor, Stella Pak, Prince Otchere

**Affiliations:** ^1^Department of Medicine, University of Texas Health Science Center at San Antonio, San Antonio, TX 78229, USA

**Keywords:** cardio-oncology, quality improvement, cardiology, oncology

## Abstract

The unattainable triangle, also known as the iron triangle or triple constraint, traditionally applied in business as a model for balancing time, cost, and quality, offers valuable insight into the field of cardio-oncology. Cardio-oncology merges cardiovascular care with cancer treatment, addressing the growing risk of cardiovascular complications in cancer patients. Similar to the business model, this specialty faces the challenge of providing timely, high-quality, and cost-effective care. The urgency of cancer treatment often strains cardiovascular assessments, while comprehensive care increases costs due to advanced diagnostics and specialized teams. Establishing a cardio-oncology center of excellence, where oncologists and cardiologists collaborate in real-time, can help balance these demands, enhance care coordination, and manage resource utilization effectively. This article explores how the specialty of cardio-oncology could deliver comprehensive, timely, and affordable patient care by applying the unattainable triangle method.

## INTRODUCTION

The unattainable triangle (also referred to as the iron triangle or the triple constraint), is a well-known concept in the business industry, and is one of the most basic parameters by which a project’s success is measured [1]. Business literature from the 1960s and well into the 1990s taught this triangle as one of the most basic and primary ways by which a manager could measure the success of their project [2]. Traditionally, the three sides of this triangle are: time, cost, and quality. Over-extending demands on any of the vertices will strain the others. Cardio-oncology is an emerging field that bridges the gap between cardiovascular care and cancer treatment, presenting unique clinical and operational challenges. As cancer therapies increasingly intersect with cardiovascular health, healthcare organizations must navigate the complexities of providing high-quality, timely, and cost-effective care. As in any successful business model, these three components need to be well balanced. In cardio-oncology, patients undergoing cancer treatment are at a heightened risk for cardiovascular complications, requiring comprehensive monitoring and management from both oncologists and cardiologists. This dual focus on oncology and cardiovascular health increases treatment costs due to the need for advanced diagnostics, specialized care teams, and cutting-edge therapies. Additionally, the urgency of cancer treatment often creates time constraints that can compromise cardiovascular care, while delayed heart assessments can jeopardize cancer treatment outcomes. For healthcare organizations, managing these complexities while maintaining profitability is challenging, especially given the high resource utilization and the need to balance financial viability with patient care. A thorough understanding of each of these vertices is imperative in establishing multidisciplinary cardio-oncology center of excellence, where cardiologists and oncologists collaborate in real-time to develop comprehensive care plans, reduce redundancies, improve coordination, and ensure that patients receive timely, high-quality care, while ensuring a reliable path for revenue generation [1].

While primarily being a business concept, the unattainable triangle proves applicable in other systems. Vetter et al. introduced a variant of this triangle in the surgical field called “The Triple Aim”. Its three vertices involved improved individual experience of care, improved health of populations, and reduced per capita costs of care as the three vertices. It highlighted the need for an “integrator” to serve as a mediator between these three factors, very similar to Patient-Centered Medical Homes (PCMH) in Primary Care that could bear the responsibility of balancing the needs of these three components [3]. Corroborating this claim are research articles which have shown that integrators in primary care or surgery, such as PCMHs or Perioperative Surgical Homes, respectively, have a positive impact in patient care with potential reduction of costs [4, 5].

Articles such as those mentioned above show the need to expand the idea of the unattainable triangle to other specialties in medicine. One of these fields is cardio-oncology. As newer and more effective chemotherapeutic and radiotherapeutic methods have been developed in recent years, resulting in decreased mortality among cancer patients, long term cardiac-toxicities in these patients have concomitantly risen. Cancer Therapy-related Cardiovascular Toxicity (CTR-CVT) is now the leading cause of morbidity and mortality in cancer survivors [6]. It is for this reason that the field of cardio-oncology, which seeks to prevent and treat cardiac-toxicities, has sprung up as a relatively new, and rapidly evolving, specialty within medicine [6]. Given its recency as a field within medicine, larger abstract articles on cardio-oncology’s overall improvement as a specialty are few. However, it is precisely because of its evolving nature that research into improving the field as a whole is important to provide guardrails to encourage the specialty to remain balanced with respect to improving quality in a cost-, and time-effective manner.

## THIS ARTICLE

In the recent past, significant stressors have been leveraged on to the medical community, with doctors and other medical staff often experiencing the peak of this stress. These two primary stressors include the recent COVID-19 pandemic and soaring rates of inflation. Following the peak pressure of the pandemic, polls showed that that 37% of physicians perceived themselves as being overworked. These rates for other medical staff members were even higher at almost half (47.4%). What makes these statistics particularly concerning are the strong correlations found by researchers between perception of burnout and intent to leave the workforce [7]. As of July 2022, the post-pandemic healthcare workforce had lost 176,000 workers compared to pre-pandemic levels, making these findings particularly alarming [8]. By 2025, it is predicted that there will be a 50,000–80,000 person shortage in physicians [9]. Adding to this stress is the growing burden of inflation on an already burdened healthcare system. A May 2024 report by the American Hospital Association found that the economy-wide inflation rates between 2021–2023, which had risen 12.4%, were met with only a 5.2% increase in medical care reimbursements during that same time [10]. The strained financial impact that this has on the hospital is inevitably shifted on to physicians to provide more revenue for the hospital. This increased stress on the healthcare workforce provides ample justification for research into improvement on the overall healthcare industry through the lens of such concepts as the unattainable triangle. In this article, we will seek to discuss how the unattainable triangle can, and should, be applied to the field of cardio-oncology as a growing specialty in medicine, focusing on the three vertices of improved patient quality of care, reduced costs, and timely treatment.

## COST

As newer and more effective methods for cancer treatment have emerged, resulting in improved survival rates in cancer patients, the concomitant risk of long-term adverse effects has increased. Cardiovascular complications are now one of the leading causes of death in survivors of breast and colorectal cancer approximately 10 years after their diagnosis of cancer [11, 12]. However, improved outcomes have been shown through increased knowledge and monitoring of CTR-CVTs. As an example, congestive heart failure rates in patients exposed to anthracycline-based treatment have declined from estimates as high as 26% to less than 3% in the course of approximately five years with improved knowledge and interventions such as minimizing the dosing of the anthracycline drug [13]. Improvement in monitoring and treatment of cardiac conditions comes at an increased cost. While the specific costs of CTR-CVTs are relatively unknown, in 2014 it was estimated that the average cost of medical care for a male cancer survivor was $4,187 per patient per year [14]. Exacerbating these concerns is the fact that many treatments for cardiovascular-related conditions are not covered by insurance. Examples of these services include biomarker testing during treatment, post-radiation non-invasive cardiac testing, cardiac magnetic resonance and strain imaging [15].

There have been a series of recommendations made to improve costs in the cardio-oncology field which we will seek to summarize here. One of the recommendations for improving costs is through a comprehensive Cardio-oncology rehabilitation program, which is a potential solution that may reduce the burden of severe cardiovascular complications from cancer therapy [16, 17]. Additional recommendations for improving costs were discussed in a recent article from 2020 in the BMC Journal of Cardio-Oncology, which collected data on the establishment of a successful and cost-effective cardio-oncology program. Four fundamental elements were discussed in this article that highlight a comprehensive way for other institutions to develop similar programs that can subsequently decrease the overall costs of cardiological disease management in the field of oncology [15]. First is the creation of a multidisciplinary team with the integration of staff and resources from the cardiac and vascular specialties into cancer centers. This was achieved by offering cardio-oncology services at both the Cancer Center as well as the Heart and Vascular Center, which allowed for interaction of the cardio-oncologists with staff from both centers. Second was the importance of continued education for members of this evolving field. This was done by encouraging a collegial atmosphere through lectures and educational workshops for staff and trainees. The third element was engagement of the cardio-oncology physicians with professional societies such as the ACC Cardio-Oncology Advocacy Work Group and the Florida Chapter of ASCO. The fourth and final element was the development of a robust research program through data collection modalities and cooperation with other specialties (namely, oncology) and other institutions [15]. These techniques have potential for long-term positive impact in the cost of care for cardio-oncology patients.

## QUALITY

Quality of care and cost of management are often inter-linked particularly in the field of cardio-oncology as many of the surveillance mechanisms and rehabilitation programs discussed earlier derive their primary cost benefit by prevention of major cardiovascular complications. One of the biggest factors to improve the quality of care in the field of cardio-oncology is development of multi-disciplinary teams that can handle the comprehensive management of this niche, but expanding, group of patients. A study conducted in the Netherlands from 2022 showed that over half of cardiologists, and nearly one-third of hematologists and medical oncologists, were unaware of their specialty’s guidelines for the management of cardio-toxicity. Over 60% of specialists in the fields of cardiology, radiation oncology, and medical oncology all expressed a desire to improve their awareness of cardio-oncology [18]. This highlights a relatively simple, and yet highly effective way to dramatically improve the quality of care in this field. An additional area of quality improvement in this field is appropriate monitoring. While ejection fraction (EF) in many ways is the gold standard of detecting heart function, it is only able to detect irreversible damage that has already been exacted on the heart. In a field such as oncology where damage to the body must be measured in real-time, the importance of subclinical markers for monitoring is vital. It is for this reason that research into such testing methods as heart strain is vitally important. While biomarkers such as troponin and B type natriuretic peptide can be helpful in this regard, they still fall short of being prognostic in their value to a specific patient [6]. Research, however, is being conducted on newer biomarkers such as interleukin-6, C-reactive protein, myeloperoxidase, Galectin-3, and growth differentiation factor-15 and their ability to detect earlier evidence of cardiac damage [19].

## TIME

The final and perhaps most complex vertex of the unattainable triangle as it relates to the other two vertices of the triangle is time. While cost and quality can be analyzed similarly, it becomes more challenging to examine timing of care. Given the medical complexity of this population due to the broad diagnoses of cancer, it becomes difficult to individualize optimal timing of care for every malignancy and CRT-CVT. Ideally, patients at an elevated risk of developing CRT-CVT should be identified early on in order to reduce morbidity and mortality as well as reducing health care costs such as decreased hospitalizations. Additionally, timing for appointments and routine follow-up studies become difficult to individualize by malignancy. Time is perhaps the most immediate and noticeable concern for a patient. Increased monitoring standards create a burden on patients. It can often become a challenge for patient compliance as long-term benefits might not be perceived by patients. More often than not, patients with advanced disease or sub-optimal support system at home can face challenges in attending appointments due to transportation issues or health related limitations. This leads to worse quality of care as several studies have shown that lack of appointment adherence leads to worsened outcomes [20–22]. Adding to this stress is the fact that each additional visit that a cancer patient makes can be a significant burden of time out of their routine activity. Cancer patients, on average, spend more than 190 minutes for ambulatory outpatient appointments and more than 270 minutes if these appointments include additional lab work and infusion therapy. Patients with advanced solid tumors may spend up to a quarter of their days alive in hospital visits or in other healthcare-related scenarios. These already strenuous time burdens on patients must be considered as new guidelines and treatments are being created in the field of cardio-oncology [23]. However, the taxing burden of time from clinic appointments and treatments in cancer is not merely restricted to the patients but can place a significant burden on clinicians as well. These considerations are particularly important in fields such as cardiology and oncology where rates of burnout reported by physicians can be as high as 50% and 59%, respectively [24, 25]. Factors that have been suggested as contributing to this phenomenon are increased burden work and complexity of treatment in medicine as well as the demand from the larger administration of hospitals for physicians to see more patients in a shorter amount of time [26]. These factors point to the importance of balancing time constraints for both physician and patient wellbeing.

While research specifically on improving time management in the field of cardio-oncology is relatively lacking, many articles that have studied improved time efficiency in other fields of medicine can provide key insights to improve the workflow for patients and physicians in cardio-oncology. We will summarize a few of these articles here. A recent study by McDermott et al. in 2024 found that the implementation of an identification card system in an orthopedic hand surgery clinic could reduce the time from check-in to being roomed by clinic staff from a mean of 21 ± 19 minutes to 13 ± 13 minutes [27]. Additionally, Ramly et al. found that self-rooming was preferred by 86% of patients and was associated with reduced waiting times, indicating that self-rooming can significantly decrease the time patients spend finding their office room [28]. Research into newer technology, such as augmented intelligence (AI) has likewise shown promise for improving time management. In a study by Li et al., the median waiting time for patients using an AI-assisted system was 0.38 hours compared to 1.97 hours for those using conventional methods [29]. However, it is not merely the patient’s time efficiency that must be considered, but the physician’s as well, as this is often the root cause of time delays in the clinic and hospital. Some of the recent articles that have studied this topic include a recent article by Chi et al., which showed that an AI system designed to organize and display new patient referral records could significantly reduce the time physicians spent reviewing electronic health records (EHRs). With their AI model, first-time users saved 18% of the time required to answer clinical questions compared to standard methods, without any statistical compromise in accuracy [30]. While individually these changes may not have significant impacts in time management, when compounded across several patients over several days, the changes hold promise to significantly reduce the time burden on both patients and physicians.

## SOLUTIONS FOR MANAGEMENT OF THE UNATTAINABLE TRIANGLE

Though improvements to the individual vertices of the unattainable triangle are mentioned previously in this article, these suggestions do not fully encompass the difficulty of managing these problems as a whole unless the triangle is observed and managed with each of the vertices in mind. As an example, if a new biomarker to measure cardiac stress were developed, as was previously suggested, this would undoubtedly improve quality, but may do so at the cost of higher prices to the patient and longer wait times for lab results. These vertices must always be viewed in light of one another if adjustments to the healthcare system as a whole are to be successful.

Suggestions have been made in the past to manage the triangle as a whole. The previously mentioned perioperative surgical home (PSH), modeled after the idea of the PCMH, looks to the role of an integrator who can manage all three vertices simultaneously. The justification behind this choice is related to the idea that this individual can provide a better experience for the patient and improvements in quality can be made as this integrator works with a team of various surgeons, anesthesiologists, and others to develop standardized clinical assessment and management plans (SCAMPs), which can improve the quality. While this model does hold promise to improve the quality of care for an individual patient, even articles promoting the use of a PSH noted that it was limited in its ability to improve the overall cost of care [3].

## POSSIBLE REBUTTALS

Some may suggest that achieving a perfect balance of the unattainable triangle is relatively impossible. Thus, research into this concept and its application to various fields of medicine is a futile effort. We posit in this article that the unattainable triangle merely provides a framework by which healthcare administrators can better create appropriate goals for their individual hospitals and departments. In their article on the perioperative surgical home, Vetter et al., address this concern by appealing to hospitals to work interdepartmentally to determine which of the vertices of the triangle that the organization seeks to improve on, understanding that doing so may come at the expense of the other vertices [3]. So long as this viewpoint can be maintained by healthcare administrators and by physicians, the strain that often characterizes the relationship between these two entities can be better managed. Administration will be aware that cutting down time on patient visits may subsequently reduce quality, and thus increase costs in the long-run. Likewise, physicians can better understand that longer visit times may also create strain on the healthcare system through reduced revenue in the more immediate moment, which can restrict the hospital’s ability to provide better resources.

## CONCLUSIONS

The challenge of balancing cost, quality, and time in cardio-oncology is significant, but with the right strategies, healthcare organizations can provide optimal care for patients while maintaining financial sustainability. By adopting integrated care models, leveraging technology, and focusing on efficiency, organizations can navigate the complexities of cardio-oncology. While each of the individual vertices of the unattainable triangle can individually be improved, a truly successful approach will try to balance all of its components for better to provide the best healthcare experiences for both the patients and the provider. A short review of the current literature highlights the relative lack of discussion around the unattainable triangle in medicine, and those articles that do discuss it often highlight one or two, but rarely all three of the vertices of this important triangle. While a perfect model for managing the unattainable triangle may be simply that, “unattainable”, investments in research, patient-centered care, data-driven decision-making, and financial alignment with payers will be crucial to the long-term success of both patient outcomes and the organization’s profitability.
